# Supplementary material for: Distribution, epidemiology, and antimicrobial resistance pattern of gram-negative bacteria isolated from blood: A retrospective study in a tertiary care hospital, Dhaka, Bangladesh
Source: PLOS Glob Public Health. 2025 Oct 31;5(10):e0004915. doi: 10.1371/journal.pgph.0004915 (PMC12578139; doi:10.1371/journal.pgph.0004915)
Supplement: Attachment — Submitted filename: Response to Reviewers.docx [file pgph.0004915.s003.docx]

**Response to Reviewers**

Thank you very much for the reviewers' and editors' encouraging comments on the manuscript entitled " Distribution, epidemiology, and antimicrobial resistance pattern of gram-negative bacteria isolated from blood: a retrospective study in a tertiary care hospital, Dhaka, Bangladesh" (Manuscript ID PGPH-D-25-01638). Those comments were very valuable, and I have made corrections point-by-point, which I hope will align with this journal's publication criteria. The revised points of the journal requirements and responses to reviewers' comments are as follows:

**Journal Requirements:**

1. Please provide additional details regarding participant consent. In the ethics statement in the Methods and online submission information, please ensure that you have specified (1) whether consent was informed and (2) what type you obtained (for instance, written or verbal, and if verbal, how it was documented and witnessed). If your study included minors, state whether you obtained consent from parents or guardians. If the need for consent was waived by the ethics committee, please include this information.

If you are reporting a retrospective study of medical records or archived samples, please ensure that you have discussed whether all data were fully anonymized before you accessed them and/or whether the IRB or ethics committee waived the requirement for informed consent. If patients provided informed written consent to have data from their medical records used in research, please include this information."

**Response:** IRB has waived the requirement of informed consent for this retrospective study of laboratory data records. All the data was anonymized before being accessed by assigning an identification number to each participant.

2. We noticed you have some minor occurrence of overlapping text with the following previous publication(s), which needs to be addressed:

-DOI: 10.7759/cureus.11000

-<https://doi.org/10.1016/j.mehy.2020.109861>

In your revision ensure you cite all your sources (including your own works), and quote or rephrase any duplicated text outside the methods section. Further consideration is dependent on these concerns being addressed.

**Response:**

-DOI: 10.7759/cureus.11000: **cited as reference 13**

-<https://doi.org/10.1016/j.mehy.2020.109861>: rephrased accordingly

3. We have amended your Competing Interest statement to comply with journal style. We kindly ask that you double check the statement and let us know if anything is incorrect.

**Response:** I have checked the competing interest statement; this seems right.

4. We note that your Data Availability Statement is currently as follows: [All the data related to this manuscript have been uploaded as Supporting Information files.]

Please confirm at this time whether or not your submission contains all raw data required to replicate the results of your study. Authors must share the “minimal data set” for their submission. PLOS defines the minimal data set to consist of the data required to replicate all study findings reported in the article, as well as related metadata and methods (<https://journals.plos.org/plosone/s/data-availability#loc-minimal-data-set-definition>).

For example, authors should submit the following data:

- The values behind the means, standard deviations and other measures reported;
- The values used to build graphs;
- The points extracted from images for analysis.

Authors do not need to submit their entire data set if only a portion of the data was used in the reported study.

If your submission does not contain these data, please either upload them as Supporting Information files or deposit them to a stable, public repository and provide us with the relevant URLs, DOIs, or accession numbers. For a list of recommended repositories, please see <https://journals.plos.org/plosone/s/recommended-repositories>.

If there are ethical or legal restrictions on sharing a de-identified data set, please explain them in detail (e.g., data contain potentially sensitive information, data are owned by a third-party organization, etc.) and who has imposed them (e.g., an ethics committee). Please also provide contact information for a data access committee, ethics committee, or other institutional body to which data requests may be sent. If data are owned by a third party, please indicate how others may request data access.

**Response:** This SPSS Statistics data set contains the raw data required to replicate all study findings reported in the article.

6. If the reviewer comments include a recommendation to cite specific previously published works, please review and evaluate these publications to determine whether they are relevant and should be cited. There is no requirement to cite these works unless the editor has indicated otherwise.

**Response:** Done accordingly.

**Reviewer report:**

1. What are the main claims of the paper and how significant are they for the discipline? Although the claim is raised in the background of the text as highlighted below, it is important that authors should highlight in the abstract section.
   Moreover, authors should draw further attention to the scarcity of epidemiological data in the study population relevant to the study settings and explain its importance by examining AMR trends/infection prevalence in the study setting.

   It is well acknowledged that AMR 77 is a worldwide concern that mostly affects low-income countries. Scrutiny of microorganism 78 distribution, BSI epidemiology, changing antibiotic resistance rates, and demographics are 79 required to support appropriate therapy. This study aimed to ascertain the prevalence, distribution, 80 and antimicrobial resistance patterns with MDR, and XDR status, of commonly isolated GNB by 81 age, sex, and hospital units through the analysis of the blood cultures of patients at Dhaka Medical 82 College Hospital (DMCH), a tertiary care hospital in Dhaka, Bangladesh.

**Response:** The main claims and significance of this study in this discipline have been included in the abstract section as follows-

Data on bloodstream infections (BSIs) and antimicrobial resistance patterns (AMR) in Bangladesh are limited. The objective of this study was to address this challenge by analyzing the prevalence, distribution, and resistance patterns—including multidrug-resistant (MDR) and extensively drug-resistant (XDR) status—of Gram-negative bacteria isolated from blood cultures at Dhaka Medical College Hospital, stratified by age, sex, and hospital unit.

2. **Materials and Methods:** Although one of the objectives of the study is to provide prevalence data, there is no clear evidence of sample size calculation or indication of the number of Blood isolates considered for the analysis.

**Response:** This was a retrospective study based on existing microbiology laboratory records over the defined study period; therefore, a sample size calculation was not performed. The samples that met the inclusion and exclusion criteria during the study period were included in this analysis.

3. Bacterial blood culture yielded 427 isolates during the study period. This section comes from the Results section. However, this information needs to be provided at the beginning, along with the number of isolated collections.

**Response:** The number of isolated collections was provided in the abstract section as follows: Total 3753 blood sample were analyzed in this timeframe amid 427 blood sample yield bacterial growth and 3326 didn’t yield growth of bacteria.

4. Further, there is no justification for the selected antibiotics for AST screening. Is it based on the local guidelines?

**Response:** The antimicrobial agents selected for AST were according to Clinical and Laboratory Standards Institute (CLSI), 2022 guideline, cited in text and reference section (15).

5. Do the data and analyses fully support the claims? If not, what other evidence is required?

**Response:**

The objectives of the study were reflected in the results section. The results were based on available laboratory records, and the data analysis was carried out accordingly to meet the aim of this study.

6. Results, discussion and conclusion;

The authors have considered an appropriate methodology and highlighted the study's limitations, but it is not apparent what is significant to the discipline.
The majority of the study area's findings are similar to those previously published; therefore, it is challenging to determine the significance of AMR trends/prevalence patterns in the study location.

The study: Distribution, epidemiology, and antimicrobial resistance pattern of gram-negative bacteria isolated from blood: a retrospective study in a tertiary care hospital, Dhaka, Bangladesh has provided the original insight in the study setting, but it overall lacks the significance of the data to the study field.
Authors can draw from published literature to strengthen and justify the claim that they are raising in the paper.

**Response:** Justification of the claim raised in this paper has been provided in the discussion section.

The study findings provide insight into the recent antimicrobial profile, which will strengthen the antimicrobial stewardship programme and guide to formulate an effective antibiogram in this tertiary healthcare setting.
